# Collaborative Online International Learning (COIL): A Teaching and Learning Experience in Nursing

**DOI:** 10.3390/nursrep14030175

**Published:** 2024-09-11

**Authors:** Marta Roqueta-Vall-llosera, Maria del Carmen Malagón-Aguilera, Gloria Reig-Garcia, Afra Masià-Plana, Eva Serrat-Graboleda, Anna Bonmatí-Tomàs

**Affiliations:** 1 Health, Gender and Ageing Research Group, Faculty of Nursing, University of Girona, 17004 Girona, Spain; marta.roqueta@udg.edu (M.R.-V.-l.); eva.serrat@udg.edu (E.S.-G.); 2 Health and Health Care Research Group, Faculty of Nursing, University of Girona, 17004 Girona, Spain; carme.malagon@udg.edu (M.d.C.M.-A.); gloria.reig@udg.edu (G.R.-G.); afra.masia@udg.edu (A.M.-P.)

**Keywords:** Collaborative Online International Learning (COIL), collaborative learning, clinical simulation, nursing education, nursing

## Abstract

Background: Collaborative Online International Learning (COIL) involves international online activities that allow the support of transversal competencies in diverse and multicultural environments without moving from home. This paper presents the learning experiences and satisfaction of undergraduate nursing students at the University of Girona (Spain) from a COIL activity involving clinical simulation in collaboration with the University of Coventry (United Kingdom). Methods: Qualitative study of content analysis. Twelve students from each of the two universities participated in the data collection process using reflective diaries. Results: The data analysis highlighted five topics related to the COIL activity involving clinical simulation: (a) initial attitudes towards the COIL activity; (b) main learning through the COIL activity; (c) positive aspects of the COIL activity; (d) weaknesses of the COIL activity and proposals for improvement; and (e) overall evaluation of the COIL activity. Conclusions: The main learning outcomes referred to by students were the relationships between transversal competencies and the skills for life, language skills, cultural skills, and more specific skills related to clinical standards. The students were most satisfied with the teaching activities and specified positive aspects and weaknesses that will add value to future versions of the activities.

## 1. Introduction

Internationalization is the deliberate process of integrating an international, intercultural, and global perspective to enhance the quality of teaching and research in university education. Its goal is to contribute to the advancement of society by cultivating responsible citizens with an intercultural outlook [1,2].

Traditionally, internationalization primarily relied on student exchanges between universities in different countries. However, this opportunity was limited to a few students due to various reasons, such as financial constraints, institutional limitations, or personal factors [3]. As a result, higher education faces the challenge of redefining internationalization and providing innovative opportunities for student mobility and study abroad programs, as well as expanding the concept of internationalization to include experiences that bridge national boundaries and take place within the home institution. This expanded approach is commonly referred to as “internationalization at home” [4,5]. Internationalization at home serves as a means to promote common values and closer understandings between different peoples and cultures, and to enhance cooperation between institutions in their internationalization efforts, while also improving the educational quality through mutual learning, comparison, and the exchange of good practice [5].

On the other hand, the world’s increasing globalization presents a challenge for nursing education, as it highlights the importance of training nursing educators to address the unique care needs of various populations, integrate international students into their programs, and develop curricula that better prepare nurses for a technologically advanced and globalized world with a focus on global health [6]. Offering nurses opportunities for international experiences and collaborations is a powerful method for inspiring them to become global citizens who can help enhance healthcare, and it fosters connectivity between local and global communities [7].

One specific initiative within the realm of internationalization at home is Collaborative Online International Learning (COIL). COIL is a pedagogical approach using digital technology to provide experiential international learning without travel abroad that enables students to develop globalized skills within diverse multicultural environments [8]. The primary objective of COIL is to enhance students’ understanding of different cultural perspectives by engaging in shared activities. This fosters a reflective dialogue among students and provides a global and multicultural dimension to the subject matter [9].

### 1.1. Clinial Simulation in Nursing Studies

At present, clinical simulation is widely recognized as an effective teaching strategy that enhances the development of nursing students’ competencies [10,11,12]. It involves recreating scenarios that mimic real-life situations to provide opportunities for practice, learning, evaluation, testing, and knowledge acquisition related to systems or human actions [13]. The fidelity of clinical simulation, which refers to the extent to which it resembles reality [14], can be categorized into six levels ranging from low fidelity to very high fidelity. Furthermore, according to Miller’s framework [15], skill acquisition is best facilitated through a progression of phases: knowing the skill, knowing how to perform the skill, demonstrating the skill in simulated cases, and applying the skill in practice. In line with this, clinical simulation requires debriefing, which is a crucial element of the process. Debriefing provides an opportunity for analysis and reflection on the clinical scenario, thus enhancing learning [16,17].

The benefits of student learning through simulation have been well documented [18,19]. Additionally, the value of COIL in fostering the acquisition of competencies among university students has been recognized [8,20]. However, there is currently a lack of research evaluating student learning through simulation conducted via COIL. In 2022, the nursing faculties of the University of Coventry and the University of Girona developed a proposal to conduct a clinical simulation COIL activity. The objective of this paper is to assess the learning outcomes of second-year nursing students from these two universities who engaged in clinical simulations through a COIL activity.

### 1.2. Description of the Organization of the COIL Activity

**Figure 1:**
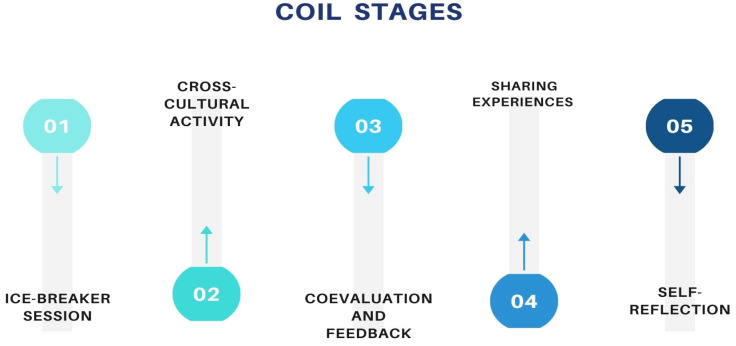
Development stages of the COIL.

The COIL activity proposed in this study consisted of five stages where students of both universities worked together in groups simultaneously: (1) an icebreaking activity, (2) group-based clinical simulation activities, (3) co-evaluation and feedback, (4) experience sharing, and (5) self-reflection on individual learning (Figure 1). Some of these activities were conducted synchronously, involving students from both universities, while others were asynchronous. Additionally, nursing teachers participated in certain activities throughout the COIL program.

Prior to initiating the COIL activity, groups were created consisting of three second-year nursing students from the same university. These groups worked collaboratively throughout all the activities proposed within the COIL framework.

The first stage of the COIL activity was the icebreaking session. This synchronous session lasted approximately 60 min, during which students were encouraged to share cultural traditions from their respective countries. Teachers and researchers played a role as moderators in facilitating this discussion. Following the icebreaking session, the second stage involved conducting the clinical simulation activity in groups. In this stage, students were instructed to prepare and self-record their resolution of one of the two low-fidelity simulation scenarios specially designed for the project. The students had the option to choose between two scenarios to be performed in the lab: the intramuscular puncture scenario, in which they were required to perform the technique on a mannequin, or the sterile field preparation scenario, where they were tasked with preparing a sterile field for male bladder catheterization. Fourth-year nursing students provided support and guidance only during this stage. The clinical simulation scenarios performed in the lab were recorded and managed using the CAE-Learning-Space Clinical Simulation^®^ [21], which was the platform used in the nursing program at the University of Girona.

The third stage of the COIL activity involved the co-evaluation of the scenario resolution. Second-year students reviewed a video of another group’s scenario recorded on the CAE-Learning-Space Clinical Simulation^®^ platform and evaluated the scenario using a standardized rubric incorporated in the same platform. The fourth stage aimed to foster the sharing of experiences among students from the different universities. A synchronous session was organized between the students from Girona and Coventry for this purpose. During this session, teachers and researchers assumed the role of observers. The final stage of the COIL activity involved writing reflective individual learning diaries, where students were encouraged to reflect on their personal experiences and insights gained throughout the COIL process.

## 2. Methods

### 2.1. Design

The study conducted was a qualitative descriptive design study of students’ reflections recorded in individual reflective journals. The analysis was approached from a naturalistic constructivist perspective. In constructivism, reality is viewed as an interpretation of the world. It acknowledges that there can be multiple realities, as each interpretation contributes to the creation of knowledge by highlighting common characteristics among different interpretations [22].

### 2.2. Sample Recruitment and Data Collection

For the COIL activity, all second-year nursing students were invited to participate. An invitation was sent in an email from the academy secretariat to ensure that the recruitment process was independent of the teaching staff. Interested students who wished to participate voluntarily informed the academic secretary, who subsequently provided this information to the researchers. The sample for this study was selected using consecutive sampling based on the order of enrolment in the activity, resulting in a total of 12 participants.

Data collection was carried out through reflective journals focused on the participants’ learning experiences during the COIL activity. The content of these self-reflections was based on the framework proposed by Colomer et al. [23], which consisted of four components exposed in the student’s reflective diary:

(a) Self-knowledge: Participants described their perceptions, attitudes, emotions, and reactions during the COIL activity.
(b) Learning experience: Participants contrasted their prior knowledge and stereotypes about other cultures, commented on their participation and collaboration within the group, and identified both facilitating elements and challenges encountered during the activity.
(c) Self-reflection in the learning process: Participants identified positive and negative aspects of the COIL activity, pinpointed areas for improvement, and discussed the potential personal and professional impact of their learning.
(d) Self-regulation of learning: Participants analyzed their planning strategies during the activity and described how they resolved difficulties or challenges that arose.

All the components reflected in the diary were developed autonomously in the personal reflective diary, having a timeline of one week to finish the assessment.

### 2.3. Data Analysis

The data collected from the reflective journals were analyzed using content analysis conducted by two different researchers. Content analysis, as defined by Krippendorff [24], is a research technique used to draw replicable and valid inferences from texts or other meaningful materials in relation to their contexts of use. The process of qualitative content analysis typically involves four stages: decontextualization, recontextualization, categorization, and compilation [25]. In order to enhance the validity of the findings, the themes identified during the analysis were discussed and clarified until a consensus was reached among the researchers [26].

### 2.4. Ethical Considerations

This study obtained approval from the Ethics Committee of Girona University (CEBRU0014-21; 28 May 2021) and adhered to the ethical principles outlined in the most recent version of the Helsinki Declaration and good practice guidelines, as stipulated by current Spanish legislation (Law 14/2007 on Biomedical Research).

All participants in this study were provided with both oral and written information about the research. They were assured of the confidentiality and privacy of their data. Participation in this study was entirely voluntary, and participants provided their written consent to take part.

## 3. Results

A total of 12 female students between 19 and 21 years old participated in the COIL activity. The analysis identified five themes related to the COIL activity: (a) initial attitudes towards the COIL activity; (b) main learning through the COIL activity; (c) positive aspects of the COIL activity; (d) weaknesses of the COIL activity and proposals for improvement; and (e) overall evaluation of the COIL activity. Table 1 provides a description of the themes and categories that emerged from the participants’ reflective journals on the COIL activity. The similarities in how the 12 participants experienced COIL were striking and reinforced confidence in our findings and conclusions.

### 3.1. Initial Attitude towards the COIL Activity

Some students who participated in the COIL felt excited, curious, and motivated about the activity, as it would allow them to exchange experiences and nursing practices with people from different countries and cultures.

*“…I was really excited about the opportunity to meet people from different countries.”*

[Participant 3]

*“I was curious because I didn’t know how it would unfold”*

[Participant 11]

*“When we were presented with the opportunity …I signed up. I was motivated to exchange experiences and common practices with people from other countries, in this case, England.”*

[Participant 1]

They also considered it interesting because it would allow them to develop and improve their communication skills.

*“…I saw it as an opportunity to develop communication skills.”*

[Participant 2]

However, some of them felt uncertain because of the language and poor familiarity with COIL activities. The main barrier that all the Girona students faced was related to the English language. The participants mentioned having a high-intermediate level of English, but they were concerned about not being able to fully understand the participants from Coventry.

*“…I was afraid of not being able to actively participate or to follow the conversation fully.”*

[Participant 3]

*“Having to speak in a different language than our own about nursing topics didn’t seem easy to me.”*

[Participant 5]

Additionally, it was the first time that a COIL activity was proposed at the nursing faculty, so there was unfamiliarity among the students regarding this activity, and that also posed a barrier to signing up for it.

*“I had never participated…I had doubts”*

[Participant 11]

### 3.2. Main Learning through the COIL Activity

**Figure 2:**
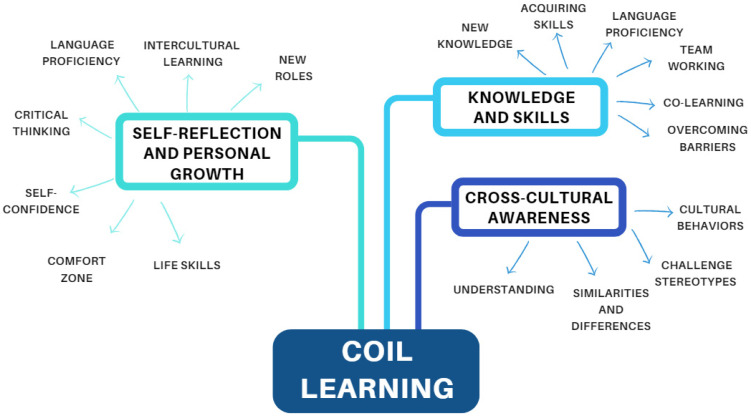
Learning themes and categories tree.

Through the reflective journal, the participants shared various things they learned from the COIL activity, as shown in Figure 2.

#### 3.2.1. Cross-Cultural Understanding and Awareness

The first lesson was cross-cultural understanding and awareness. Recognizing nursing variations across countries was a valuable outcome from COIL. Students observed similar nursing responsibilities with minor differences. They also discovered interesting aspects of nursing, like uniform variations, in other countries.

*“…I used to think that the role of nursing was the same in all countries, that it was universal. But now I have realized that it’s not the case.”*

[Participant 5]

*“I found it interesting the uniform and apron they were wearing because it is not common here to wear a uniform in a colour other than white, and it is also not common to use a protective apron like that.”*

[Participant 10]

Additionally, the participants were able to learn about the similarities and differences between nursing technique protocols in the two countries. They had the opportunity to reflect on incorporating aspects they observed in the technique of students from the other country, with the aim of improving their own technique.

*“Thanks to the implementation of COIL, we were able to observe all those aspects that we have in common with English students when performing different nursing procedures.”*

[Participant 7]

*“I have questioned the different ways of performing a technique.”*

[Participant 4]

At this same point, the participants reflected on feeling surprised, especially with the hand hygiene procedure of the English students, who repeated this procedure many more times during the same technique. The verification of materials before the procedure and the information provided to the person about the technique they would undergo were other aspects of the Coventry students that positively surprised the Catalan students. However, the majority of participants affirmed that despite the differences in care protocols, the goals and outcomes were the same.

*“During the activity, we observed that the English students followed a stricter handwashing protocol”*

[Participant 3]

*“…despite the differences between English and Catalan culture when performing the same technique, the goal and final outcome are the same.”*

[Participant 11]

The opportunity to share the activity with individuals from different cultural backgrounds was also emphasized by the participants as a valuable learning experience. This experience helped them enhance their attitude towards working with people from diverse cultural contexts. Through the COIL activity, the Catalan students were able to challenge stereotypes they held about the English, whom they had previously perceived as more formal, distant, strict, and even superior.

*“Also, this experience helped to improve my attitude towards working with different people from different cultural backgrounds.”*

[Participant 12]

*“I was expecting to encounter a very robotic, distant, and impersonal attendance (referring to the participation of the students from Coventry). To my surprise, it was quite the opposite.”*

[Participant 2]

Only one of the participants identified the behavior of the English students as being more formal and distant towards the person being served.

*“The students from Coventry keep more distance, they don’t get as close to the patient.”*

[Participant 6]

#### 3.2.2. Acquisition of New Knowledge and Skills

Through the activity, the participants were able to review nursing techniques they had studied during the previous academic year, specifically the technique of preparing the sterile field and the technique of intramuscular injection.

*“The COIL has helped us review how to prepare a sterile field and perform an intramuscular injection.”*

[Participant 7]

Furthermore, all the participants noted in their reflective journals that the COIL activity had enabled them to enhance their proficiency in the English language, particularly in terms of vocabulary, nursing-specific terminology, pronunciation, and fluency.

*“I have been able to learn and improve my English.”*

[Participant 4]

*“You have the opportunity to gain vocabulary in English regarding the materials to be used in each practice… and thus speak fluently.”*

[Participant 3]

Another learning experience that the participants considered useful in COIL was the collaborative learning and the ability to work as a team, especially when overcoming the barriers that arose during the activity. They recognized this as an essential skill for their future professional careers as nurses.

*“By sharing knowledge and working as a team, in the end, we were able to achieve it.”*

[Participant 8]

*“Where one couldn’t reach, the other would do it, we learned it together.”*

[Participant 4]

#### 3.2.3. Self-Reflection and Personal Growth

Promoting and fostering critical thinking among students was another key lesson identified by the participants from the COIL activity.

*“…I think it is a great way to encourage essential attitudes for everyday life, such as critical thinking.”*

[Participant 9]

Moreover, the students regarded their participation in the COIL activity as a catalyst for stepping out of their comfort zones, which they viewed as a valuable learning experience with personal and professional benefits. Additionally, through the activity, they had developed a greater sense of security and confidence.

*“We stepped a little further out of our comfort zone, we opened ourselves up to discover what lies beyond, and we saw areas where we could improve.”*

[Participant 9]

*“It has helped me feel confident in myself.”*

[Participant 1]

Through the activity, the Catalan nursing students also recognized the importance of being able to express themselves effectively in the English language. This motivated them to continue studying the language and participate in other nursing faculty activities conducted in English. Additionally, after participating in COIL, some students expressed their interest in taking part in an Erasmus program or even working as nurses in other countries in the future.

*“I have realized the importance of being able to express oneself well in English and of learning this language.”*

[Participant 3]

*“Engaging in this activity makes me consider the possibility of participating in Problem-Based Learning in English in the coming academic year.”*

[Participant 11]

*“Having participated in the activity has also opened doors in my mind when considering doing internships abroad or even going to work there.”*

[Participant 1]

### 3.3. Positive Aspects of the Proposed COIL Activity

The participants highly appreciated the well-organized and dynamic nature of the activity.

*“I was pleased with how it was organized and dynamized.”*

[Participant 10]

At this point, noteworthy strengths of the COIL’s organization included the ability to select collaborative peers (the workgroup), coupled with the optimal group size of three students, which greatly facilitated the seamless coordination of the various proposed activities. Additionally, the flexibility in scheduling, particularly pertaining to the choice of day and time for attending the simulation laboratory and conducting the practice recording, further enhanced the efficacy of the program.

*“I think being in a group of 3 has been useful”*

[Participant 7]

*“It has become easier with the provided timetables (referring to the schedules for recording the practice at the simulation laboratory).”*

[Participant 1]

Other notable strengths highlighted by the students were the dynamic, facilitative, and approachable roles of the professors in charge of the COIL activity throughout the various activities, particularly during the initial synchronous meeting.

*“The professors share anecdotes and facilitate student expression.”*

[Participant 8]

In addition, a respectful and trusting atmosphere was fostered throughout all the activities, both during synchronous meetings and in the group work among the students.

*“There was a climate of trust and respect, and that made me feel very good.”*

[Participant 10]

The participants also emphasized the strength of having the support and mentorship of fourth-year nursing students during the activity. They were guided specifically in the development of the laboratory practice and had the opportunity to have exchanges about other aspects of the nursing degree.

*“…the assistance of the fourth-year nursing students, as they provided us with advice that we wouldn’t have thought of ourselves. In addition to tips for making the COIL video, we also had discussions about the nursing career in general.”*

[Participant 1]

In relation to the different activities, the icebreaking activity received the highest rating in most of the reflective journals. The students found the chosen theme for this activity, cultural curiosities from each country, to be highly relevant and engaging.

*“In the icebreaker, I shared traditions from Catalonia. Sharing the traditions of your country with others is something I find very positive and a good way to get to know people and break the ice.”*

[Participant 8]

Finally, the students valued both the self-assessment and the peer assessment of the simulation practice videos, considering the evaluation as a learning opportunity. They emphasized the importance of evaluative rubrics for objective and facilitated corrections.

*“Very interesting activity as we acted as teachers and evaluated based on our own knowledge and the provided rubric.”*

[Participant 8]

### 3.4. Weaknesses of the COIL Activity and Proposals for Improvement

By analyzing their reflective journals, the students identified some weaknesses in the COIL activity and suggested possible improvements. The primary weakness observed was the inclusion of unrelated topics during synchronous meetings between students from different countries, leading to hindered focus and potential distractions.

*“They were discussing some topics that were unrelated to COIL, and I couldn’t follow them very well.”*

[Participant 1]

Having groups composed of students from the same country for the simulation practice and recording was considered a weakness. Students felt it would have been more positive and interesting to have heterogeneous groups with students from different countries, despite acknowledging the greater organizational difficulty.

*“It would have been more enriching, despite requiring more time and coordination, if the groups were composed of students from both universities.”*

[Participant 2]

At the same time, they also identified as a weakness the number of synchronous sessions conducted between students from both universities. The students expressed a desire for increased interaction with their counterparts from other countries. As a solution, they proposed augmenting the working time and session frequency between students of both countries through the implementation of research activities.

*“I wanted to have a couple more sessions with the students and professors from Coventry… it felt too short.”*

[Participant 3]

*“Incorporating some additional activities… a good option would be to have some small group assignments with students from different countries.”*

[Participant 5]

The lack of planning on the part of some students, particularly due to the COIL activity overlapping with their clinical placements and exam resits, as well as the fact that group members were from different towns and did not reside in Girona during the activity period, were also identified as weaknesses. The students proposed scheduling the COIL activity outside the clinical placements and exam period, and also suggested involving students from the same town to enhance planning and coordination.

*“I didn’t have much time to prepare the script for the video due to clinical placements and exam resits… it should have been done at another time.”*

[Participant 10]

*“Difficulty in planning arose from my group members not being in Girona to perform tasks like script preparation and editing.”*

[Participant 3]

Nursing students also identified the use of a simulated environment for the practice (nursing technique) as a weakness of the COIL activity. They believed it would be more beneficial to perform the same practice in a real environment.

*“The negative aspects… and lastly, that it was a simulation and not a real case.”*

[Participant 4]

*“I think we would learn even more if it were real.”*

[Participant 9]

The proposed techniques (intramuscular injection and sterile field) were very similar in both countries. In this regard, the participants believed it would have been more beneficial to explore more complex nursing techniques with greater regional variations.

*“I would suggest altering the technique to be performed, where there may be more differences between Catalonia and England.”*

[Participant 5]

*“I would repeat it with more complex techniques to compare the protocols of each country.”*

[Participant 8]

Finally, the majority of students felt that the activity would have been more enriching if it had been conducted in person rather than online. The students expressed that the virtual format sometimes hampered communication, and technical connectivity issues frequently arose.

*“I missed face to face contact.”*

[Participant 4]

*“It would have been easier and more profitable to be able to carry out the activities in a more face-to-face manner.”*

[Participant 7]

*“One of the negative aspects of COIL is the fact that it has to be done online, since on different occasions it makes it difficult to exchange opinions and/or understanding”*

[Participant 3]

*“The problem is that being online, there are some network errors”*

[Participant 6]

There were also compatibility issues with programs and browsers among the different students, which significantly hindered video playback. The students suggested reaching a consensus on the choice of software applications to ensure smooth execution of the activity.

*“We encountered technical difficulties throughout the activity, primarily due to issues with our devices, browsers, and video uploading.”*

[Participant 9]

### 3.5. Overall Evaluation of the COIL Activity

Participants highly valued the COIL activity, praising both its structure and the proposed activities. The students’ overall assessment was overwhelmingly positive, describing the experience as enriching, motivating, exciting, and satisfying.

*“…Is a very enriching experience.”*

[Participant 6]

*“…I found it to be a good way to motivate and encourage essential attitudes.”*

[Participant 9]

## 4. Discussion

In the context of a global approach to internationalization at home, this study explored the impact on nursing students of a COIL activity using clinical simulation methodology. The results highlighted themes related to the COIL activity: (a) initial attitudes towards the COIL activity; (b) main learning from the COIL activity; (c) positive aspects of the COIL activity; (d) weaknesses of the COIL activity and proposals for improvement; and (e) overall evaluation of the COIL activity. Before starting the COIL, some students felt excited, curious, and motivated. This motivation is particularly significant for nursing students, as noted by Edgar [27] and Abdulghani [28], who emphasized that student motivation is a crucial factor in their commitment to their studies and their overall academic performance.

The participants believed that the COIL would allow them to improve their communication skills, which are very important as nursing students and also as future nurses [26,29]. According to previous studies, good communication skills in health professionals are related to successful health outcomes [30].

Therefore, increasing the communication skills of the nurses can positively affect the interventions performed in the different care services, as the nurses feel able to perform the actions needed to obtain the results sought in their work [31].

In this study, students communicated in English as a common language, which is a limitation they perceived about being enrolled in the COIL. At the same time, a study on a COIL course stated that although both student groups shared English as a common language, the varying proficiency levels led to both groups experiencing some language barriers [32]. This being the first time a COIL was performed in the nursing faculty was also another limitation for the students who participated in it. 

Through the COIL activity, the nursing students achieved several learning outcomes without observing significant differences between the two universities. Firstly, this proposed activity increased the participants’ knowledge about nurses’ roles and characteristics in both countries, such as differences in uniforms, in some guidelines and protocols, and also in some techniques. Although the differences were few, the Spanish students were able to incorporate some aspects from the Coventry students to improve the results of their techniques. According to Limoges [33], learning how another healthcare system works also helps students to understand how they can change and improve the systems in their respective countries. And working with students from another country helps build a broader understanding of nursing as a global profession.

Learning experiences that involve encounters with different cultures are important, as this supports self-awareness and contributes to the knowledge and skills necessary to consider and integrate cultural perspectives in healthcare [34]. A study with nursing students in Hong Kong and Sweden explored the effects of online discussions to plan care for a patient from admission to discharge. These online strategies assisted students to achieve intercultural learning and to develop their identity as nurses and as learners [35]. Therefore, exposing student nurses to other cultures and combining this with specifically organized learning activities enhances awareness of how culture intersects with health and how to orient nursing care to meet individual needs within a culturally safe framework [36,37,38]. In this regard, our study highlights that through the COIL nursing students can understand how culture influences the nursing role. And according to Doutrich [39], this can prompt cultural humility and the need to further consider the individual’s needs during care. Teamwork was another skill that participants developed with the activity. This is important because equipping nurses with the necessary knowledge and skills for effective teamwork through education and training is a crucial step [40].

Moreover, this proposed activity promoted critical thinking. Critical thinking is an essential process for safe, efficient, and proficient nursing practice, and nursing education programs should adopt attitudes that promote self-reflection and mobilize the skills of critical reasoning [41]. Moreover, in support of our results, Philips [42] pointed out that international experiences and exchanges are useful learning strategies to stimulate reflection. Learning to get out of the comfort zone and increasing self-confidence were other lessons learned with COIL. Motivation to believe in oneself with learning may be influential in education [27].

Successful teamwork is recognized as an essential component of much effective health care. It reduces the stress that patients may feel, ensures their safety, and also can reduce the number of nurses’ burnout problems [43,44]. Improving language proficiency and feeling motivated to continue studying English were the last types of learning reported by the participants. In addition, the COIL aroused their interest in working abroad, which could be a problem. According to data from the General Nursing Council, Spain’s public health system needs 87,000 nurses to reach the European average [45].

Some strengths of the COIL were in the organizational structure: the flexibility to carry out some activities, the different schedules proposed for each activity, and the opportunity to choose the working group. According to Naicker [32], well-structured activities coupled with the teacher overseeing the process are important elements in COIL. Also, the climate of trust and respect that was established during the different activities was part of this, such as the icebreaker, which is a technique to melt an awkward atmosphere. Sinta [46] says that in a classroom, an icebreaker can change the situation. It can raise a learning spirit in the class. As a result, students are more motivated to learn [47]. The icebreaking activity of sharing traditions and curiosities of both countries proposed in our COIL was very well assessed by students and was considered as another strength of the project. Also part of this was having to self-evaluate their own practice, and also to evaluate that of other students. According to Neimeyer [48], self-assessment is appropriate for nursing students. On the other hand, peer assessment provides multiple sources of feedback, guides students to think critically about course content, and engages them in advanced self-assessment [9,45]. However, our results showed that it is important to have specific rubrics.

This study also detected some COIL weaknesses. The dates on which this activity took place, which coincided with the clinical practices period, the number of sessions proposed, and the fact that the group of students were from the same country were detected as weaknesses. The students would have preferred more sessions and more interaction with other university students. The study of Limoges [33] stated that after a similar activity, students were enthusiastic about their learning experiences and wanted this opportunity to be continued.

Although one of the main characteristics of the COIL is the virtual environment [46,49], some of the participants felt that a face-to-face activity would be more enriching. The fact of having some connection problems could explain this contribution.

At present, clinical simulation is widely viewed as a valid teaching strategy that promotes competence development in nursing students [10,50,51]. However, this study suggests that organizing a COIL by using clinical simulation could be a weakness. Perhaps the two nursing techniques proposed in this activity, being quite similar between the two countries, could have influenced this perception.

Finally, the assessment of this COIL was very positive, both in the structure and the activities proposed. Therefore, knowing that keeping the focus on the student is central to COIL, an effective learning system can be intentionally developed, giving students a study abroad experience without leaving home [32]. We will continue to propose COIL activities and study their impact on nursing students’ learning.

### Limitations

Although this study offers valuable insights, these are restricted by certain limitations stemming from its implementation within a particular context (nursing colleges in Spain and the United Kingdom), which may limit the applicability of the findings to different nursing education backgrounds or cultural situations. Moreover, the application of a qualitative approach restricts the generalizability of the findings to a more extensive population.

## 5. Conclusions

This study revealed that the use of clinical simulation in a COIL activity facilitated the achievement of learning outcomes related to transversal nursing skills, such as critical thinking, self-confidence, cultural competence, teamwork, and English language proficiency, in addition to enhancing clinical skills. In relation to satisfaction with the teaching activity, the students considered the activity to be satisfactory, highlighting as positive aspects the good working climate, the number of students per group, and the evaluation format. These results can guide the organization of future COIL activities.

## Figures and Tables

**Table 1:** Themes and categories identified through the content analysis of the reflective journals.

Topic 1	Topic 2	Topic 3	Topic 4	Topic 5
Initial Attitude towards the COIL Activity	Main Learning from the COIL Activity	Positive Aspects of the COIL Activity	Weaknesses of the COIL Activity and Improvement Proposals	Overall Evaluation of the COIL Activity
Excitement	Cross-cultural understanding and awareness	Organization and dynamics	Inclusion of unrelated topics	Enriching
Motivation	Personal growth and self-reflection	Professors’ role	Mixed-country student groups could improve groups composed of students from the same country	Motivator
Interest	Acquisition of new knowledge and skills	Respectful and trusting atmosphere	Few synchronous sessions	Exciting
Uncertainty		Mentorship of fourth-year nursing students	Lack of contact that could be improved by sharing research projects	Satisfactory
Curiosity		The theme for the icebreaking activity	Lack of student planning	
		Self-assessment and peer assessment	Students residing in different localities and timing of the activity, which could improve if the students reside in the same town and scheduling outside internship period	
			Simulated case. The activity could improve if a real case was proposed instead of a simulated one	
			Similar nursing techniques between the two countries It could be improved by proposing different and more complex techniques	
			Online format. The experience would improve with face-to-face interaction Technical problems and incompatibility between e-learning platforms	

## Data Availability

The data presented in this study will be available on request to the corresponding author.

## Public Involvement Statement

No public involvement in any aspect of this research.

## Guidelines and Standards Statement

This manuscript was drafted using the SRQR [52] guidelines for qualitative studies.
